# Combination treatment optimization using a pan-cancer pathway model

**DOI:** 10.1371/journal.pcbi.1009689

**Published:** 2021-12-28

**Authors:** Robin Schmucker, Gabriele Farina, James Faeder, Fabian Fröhlich, Ali Sinan Saglam, Tuomas Sandholm

**Affiliations:** 1 Machine Learning Department, Carnegie Mellon University, Pittsburgh, Pennsylvania, United States of America; 2 Computer Science Department, Carnegie Mellon University, Pittsburgh, Pennsylvania, United States of America; 3 Department of Computational and Systems Biology, University of Pittsburgh, Pittsburgh, Pennsylvania, United States of America; 4 Department of Systems Biology, Harvard Medical School, Boston, Massachusetts, United States of America; 5 Strategy Robot, Inc., Pittsburgh, Pennsylvania, United States of America; 6 Optimized Markets, Inc., Pittsburgh, Pennsylvania, United States of America; 7 Strategic Machine, Inc., Pittsburgh, Pennsylvania, United States of America; Cleveland Clinic, UNITED STATES

## Abstract

The design of efficient combination therapies is a difficult key challenge in the treatment of complex diseases such as cancers. The large heterogeneity of cancers and the large number of available drugs renders exhaustive *in vivo* or even *in vitro* investigation of possible treatments impractical. In recent years, sophisticated mechanistic, ordinary differential equation-based pathways models that can predict treatment responses at a *molecular* level have been developed. However, surprisingly little effort has been put into leveraging these models to find novel therapies. In this paper we use for the first time, to our knowledge, a large-scale state-of-the-art pan-cancer signaling pathway model to identify candidates for novel combination therapies to treat individual cancer cell lines from various tissues (e.g., minimizing proliferation while keeping dosage low to avoid adverse side effects) and populations of heterogeneous cancer cell lines (e.g., minimizing the maximum or average proliferation across the cell lines while keeping dosage low). We also show how our method can be used to optimize the drug combinations used in *sequential* treatment plans—that is, optimized sequences of potentially different drug combinations—providing additional benefits. In order to solve the treatment optimization problems, we combine the Covariance Matrix Adaptation Evolution Strategy (CMA-ES) algorithm with a significantly more scalable sampling scheme for truncated Gaussian distributions, based on a Hamiltonian Monte-Carlo method. These optimization techniques are independent of the signaling pathway model, and can thus be adapted to find treatment candidates for other complex diseases than cancers as well, as long as a suitable predictive model is available.

## Author summary

Combination therapies are a promising approach to counter complex diseases such as cancers. Two key difficulties in the design of effective cancer combination therapies are the large number of available drugs and the heterogeneity of cancers which render exhaustive laboratory studies impractical. In recent years, sophisticated signaling pathway models that can predict responses to combination treatments at a *molecular* level have been developed. This motivates the question of how one can leverage mechanistic models to identify candidates for novel combination treatments. In this paper we propose a combination treatment optimization framework which employs a large-scale pan-cancer pathway model. We formulate treatment optimization problems for single cell lines and heterogeneous populations of cancer cells. We further investigate sequential treatment plans and combine an existing evolutionary algorithm with an efficient Hamiltonian Monte-Carlo based sampling scheme. During extensive simulation studies our approach identified combination therapies which are predicted to be more effective than conventional treatments. We hope that one day *in silico* experiments will be used to identify a small set of promising treatment candidates which can then form a starting point for laboratory studies, allowing for an efficient use of limited resources and accelerated discovery of effective therapies.

## Introduction

Rational design of combination therapies is a difficult but important challenge in the treatment of complex diseases such as cancers [1–6]. The large heterogeneity of cancers and number of available drugs renders exhaustive *in vivo* or even *in vitro* investigation of treatments impractical. Accordingly, computational models that enable—even individualized—prediction of drug sensitivity have to be employed [7]. To this end, sophisticated mechanistic, ordinary differential equation (ODE) models for drug sensitivity prediction have been developed [8–14]. However, so far little effort has been put towards using these models to actually design treatments. Typically, only the temporal aspect of when to administer drugs [15, 16] is considered, but not which drugs to pick.

The selection and prioritization of anti-cancer drugs needs to balance decrease in proliferation in cancer tissue with adverse side effects in healthy tissue. The anti-proliferative effect can be enhanced by using searching for synergistic drug combinations where the combination of drugs decreases proliferation more effectively that individual drugs, according to some null model such as Bliss independence [17] or Loewe additivity [18]. The higher efficacy of synergistic drugs enables these drugs to be employed at lower drug concentrations, which may reduce adverse side effects. In the cancer context, single drug efficacy has been exhaustively characterized in large databases such as the Cancer Cell Line Encyclopedia [19]. The previously mentioned ODE models can extrapolate single drug measurement to combinations and predict synergy, which is difficult to explore experimentally [9]. However, there is no comprehensive characterization of adverse effects in healthy tissues. Cells in healthy tissue are often terminally differentiated and, accordingly, do not proliferate. This makes it intractable to generate cell counts required for comprehensive *in vitro* screens and therefore adverse effects have to be investigated *in vivo* in expensive animal experiments or clinical trials.

Besides adverse effects, drug resistance is a major concern for treatment design. Drug resistance can either be innate, or acquired as a response to treatment [20]. While bio-marker guided patient stratification is possible in select cases, invasive biopsies are required to evaluate the presence of molecular markers in solid tumors. Such biopsies are expensive and only provide a limited view of clonal substructure. The issue of heterogeneity in drug resistance can also be addressed by the use of combination treatments, as the chances for susceptibility increase with the number of employed drugs [21]. This inherently also addresses the issue of acquired resistance, as drug combinations can counteract clonal selection that is frequently observed in single-drug treatments.

In this paper we present a framework for *in silico* combination treatment optimization which employs a large-scale mechanistic pan-cancer pathway model [9]. A robust evolutionary optimization algorithm is modified with an efficient sampling scheme and used to guide the search for effective drug combination. An extensive simulations study shows how different regularization strategies can be used to identify a set of combination therapy candidates—trading off low proliferation with adverse side effects—targeting homogeneous and heterogeneous tumors. Furthermore, we show how our method can be used to optimize *sequential treatment plans* which apply varying drug cocktails in sequence to prevent inherent and acquired drug resistance. The framework can be easily adapted to find treatment candidates for complex diseases other than cancers, as long as a suitable predictive model is available.

To our knowledge, this is the first application of a large-scale pan-cancer pathway model to search for novel combination therapy candidates. We adapt non-convex optimization techniques and use an efficient parallelization scheme which enables the analysis of dozens of cell lines and combinations of 7 anti-cancer agents at low cost. Three different treatment scenarios targeting single as well as multiple cell lines at once are formalized as optimization problems and simulations studies are conducted. Our simulations identified a set of treatment candidates in the form of drug combinations that achieve better predicted treatment effects at lower concentrations than the conventional therapy approaches.

### Related work

The use of mathematical modeling for the design of cancer therapies has a rich history. Early studies combined optimal control theory with a growth model of bone cancer to find treatment regimes which balance reductions in cell population with administered dosage of a single drug [22, 23]. Moreover, evolutionary game theory [24, 25] was used to analyze the adaption of cell populations under selective pressure, especially with regards to population size [26–28], and emergence of drug resistance [29–31].

Sandholm [32, 33] proposed modeling treatment planning—and steering biological entities more generally—as a multi-step game between a biological entity and a treater, for the purposes of computationally constructing steering plans that can involve combination therapies, sequential plans, and conditional plans (aka. adaptive treatments). He proposed modeling the biological entity in the game 1) using a behavioral model if there is enough data, 2) as a game-theoretic worst-case adversary if there is not enough data, or 3) as an opponent with limited lookahead so it can be exploited by luring it into traps. (Specific algorithms have since then been developed for exploiting an opponent’s limited lookahead in imperfect-information games [34, 35], but they have not yet been applied to biological settings.) In that taxonomy, the present paper falls under approach (1).

Adaptive treatment regimes [36]—that is, regimes that monitor tumor development and use predictive models to adapt reactively—have led to promising preclinical trials on breast cancer [37] and Phase 2 clinical trials on prostate cancer [38]. Multiple *in vitro* studies [39–41] investigated the emergence of drug resistance and showed advantages of adaptive treatment regimes. A recent line of work [16, 42, 43] investigates benefits of combination treatments on the development of drug sensitivity. Stackelberg games have been used in computational studies to design vaccines that impede virus adaption [44] and have more recently been proposed for cancer treatment design [45] with the motivation to control drug resistance.

While these prior approaches rely on rather high-level abstractions of the underlying biology, our work employs a detailed, mechanistic pan-cancer signaling pathway model [9]. It can be individualized to cell-lines using sequencing data, which is important to account for heterogeneity in response. It describes the action of 7 small molecule inhibitors, which enables the design of higher-order combinations. One advantage of mechanistic pathway models over other machine learning based techniques [46, 47] is that the domain knowledge encoded into their graphical structure makes them less prone to overfitting and can help with generalization. Previous evaluations of the pathway model we used indicated that it is capable of predicting the effect of drug combinations from single drug treatments with quantitative accuracy [9], which is essential for the reliability of treatment strategies we propose. The only prior work [48] in this direction uses a Boolean T-cell signaling pathway model [49] which yielded—due to its Boolean nature—mainly qualitative insights.

Our work serves as a proof of concept of how biologically accurate quantitative signaling pathway models can be combined with optimization algorithms to discover effective combination therapies, including multi-step ones. Our methodology and computational approach enabled us to perform extensive simulations for combinations of 7 existing anti-cancer agents on dozens of cancer cell-lines yielding promising directions for future laboratory studies.

## Methods

In this section we present our approach in detail. We first discuss the pan-cancer signaling pathway model that is used to simulate treatment responses. Building on the predictions of this model, we propose three different regularization strategies that minimize drug concentrations and introduce three treatment optimization problems that account for different levels of biological complexity. In order to tackle these problems we discuss modifications to the CMA-ES algorithm [50], to make it suitable for our constrained search space. Finally, we discuss simulated cell lines and combination treatments as well as implementation details.

### Pan-cancer cell simulation

For our treatment optimization simulation study, we employed a pre-existing large-scale mechanistic pan-cancer signaling pathway model [9]. The model describes the effects of 7 targeted anti-cancer agents on multiple cancer-associated pathways at the molecular level as an ODE model. In total, the model describes the temporal development of 1228 different molecular species, that is, concentrations of ligands, protein complexes or drugs, through 2704 reactions using a total of 4104 parameters. Every model simulation reports a proliferation score

$$\begin{array}{c} R\left(\tau ,e\right)=f\left(x_{ss},w\right),\;s.t.\;\dot{x}=g\left(x,p,e,\tau \right)\;\text{and}\;g\left(x_{ss},p,e,\tau \right)=0, \end{array}$$ (1)

where $f\left(x,w\right):R_{\geq 0}^{1228}\times R_{\geq 0}^{16}\to R_{\geq 0}$ is a phenomenological function that maps molecular abundances to proliferation scores, ***x***_*ss*_ are molecular abundances defined by the steady state of the ODE model and $w\in R_{\geq 0}^{16}$ are mapping coefficients, which are free parameters of the mapping function. Here, $g\left(x,p,e,\tau \right):R_{\geq 0}^{1228}\times R_{\geq 0}^{4088}\times R_{\geq 0}^{144}\times R_{\geq 0}^{7}\to R^{1228}$ is the right hand side of the differential equation. $x\in R_{\geq 0}^{1228}$, the kinetic parameters $p\in R_{\geq 0}^{4088}$ are biophysical rate constants such as binding rates or catalytic activities, which are free parameters for the ODE model. $e\in R_{\geq 0}^{144}$ are mRNA expression levels for 108 different genes and 36 gain of function mutations described by the model, which can be used to individualize the model to specific cell lines. $\tau \in R_{\geq 0}^{7}$ are drug concentrations, which define the concentrations of individual drugs in the extracellular compartment.

To be biologically meaningful, the proliferation score *r* has to be normalized to the proliferation score for the untreated condition with ***τ*** = 0. The normalized relative proliferation score *V*(***τ***, ***e***) = *R*(***τ***, ***e***)/*R*(0, ***e***) can be directly compared to experimental observations from cell viability assays such as CellTiter-Glo [51], which quantify the difference in cell counts between treated and untreated conditions, thus accounting for the net sum between cell growth and cell death.

For all simulations, we used previously reported values for ***p*** and ***w***, which were obtained by training the model on relative proliferation data from 120 cell lines from the Cancer Cell Line Encyclopedia [19]. We used the Advanced Multilanguage Interface for CVODES and IDAS (AMICI) software package [52, 53]—which internally uses a C implementation of the *Variable Coefficient ODE Solver (VODE)* [54] *called* CVODES [55]—to solve the ODE model (that is, the signaling pathway network variables) to steady state after each treatment. Default AMICI integration and steady state tolerances were used.

### Combination treatment optimization

We leverage the pan-cancer pathway model to identify candidates for novel combination therapies for a variety of cancers using 7 preexisting drugs. Formally, we represent a multi-drug treatment by a 7-tuple $\tau \in R_{\geq 0}^{7}$. Entry *τ*_*i*_ is the concentration of the *i*-th drug contained in treatment ***τ*** in nanomoles (nM). Mathematically, the set of treatments considered in this paper is represented by $T=\{\tau \in R_{\geq 0}^{7}:\|\tau {\|}_{1}\;\leq \alpha \}$, that is, the set of all combination therapies whose total dosage is below threshold value *α*. In prior work [9], the pathway model had been fitted with clinical data administering concentrations in the range from 2.5 nM to 8000 nM. Thus, we use a value of *α* = 8000 to ensure that the optimization domain T resembles the training data in terms of total dosage.

An effective treatment needs to trade off between desired and adverse effects. For each cell line *c* the model defines a function $V_{c}:T\to R_{\geq 0}=V\left(\tau ,e_{c}\right)$, which given a treatment τ∈T and a vector of expression levels ***e***_***c***_, predicts the relative proliferation value of *c* when subjected to *τ*. The predicted relative proliferation is used to capture desired treatment effects. Because the literature does not offer a concise way to quantify adverse effects on healthy cells caused by a combination of multiple drugs, we apply a mathematical regularization function $R:T\to R_{\geq 0}$ to the treatment vector as an idealized measure. Prior work has used L1 [22, 23] and L2 [56] regularization for this purpose. In our simulation study we use L1 (*R*_*L*1_), L2 (*R*_*L*2_) and sum of logs regularization (*R*_*ln*_) defined as

$$\begin{array}{c} R_{L1}\left(\tau \right)=\sum_{i=1}^{7}\left|\tau_{i}\right|,\;R_{L2}\left(\tau \right)=\sqrt{\sum_{i=1}^{7}\tau_{i}^{2}},\;R_{ln}\left(\tau \right)=\sum_{i=1}^{7}\text{ln}\left(1+\tau_{i}\right) \end{array}$$ (2)

and compare differences in resulting treatments. The following three subsubsections, respectively, introduce three different treatment optimization problem classes that are addressed in our simulation study.

#### Optimizing the single-step treatment of a single cell line

First, we focus on identifying a treatment τ∈T that is effective for a specific cell line *c*. An optimization problem which balances relative proliferation score and adverse effects is given by

$$\begin{array}{c} \underset{\tau \in T}{\text{min}}\;V_{c}\left(\tau \right)+\lambda R\left(\tau \right), \end{array}$$ (3)

where the penalty parameter λ sets the weight of adverse effects as quantified by regularizer *R* ∈ {*R*_*L*1_, *R*_*L*2_, *R*_*ln*_}. Large values of λ favor conservative treatments administering small dosages while low values favor more aggressive treatments administering larger dosages. Solving the optimization problem for a range of different penalty values results in combination treatments that are Pareto efficient with respect to their treatment effect/adverse effects profiles. The Pareto front formed by these treatments can then in principle be used as an aid in the treatment selection process.

#### Optimizing the single-step treatment of a population of cell lines

Tumors often feature multiple sub-clones that feature different sets of mutations and expression levels. To avoid resistance from clonal evolution, all possible sub-clones have to be targeted effectively. As a proxy for these sub-clones, we consider multiple cell lines with the same tissue of origin. Accordingly, we try to construct treatments τ∈T that are simultaneously effective on a set of different cell lines C. The optimization problem is

$$\begin{array}{c} \underset{\tau \in T}{\text{min}}\;\underset{c\in C}{\text{max}}\;V_{c}\left(\tau \right)+\lambda R\left(\tau \right), \end{array}$$ (4)

where the objective function only considers the highest predicted proliferation value following treatment *τ* among the cell lines in C, that is, the most proliferated cell line. This objective favors treatments that reduce the proliferation values of all cell lines in the population evenly.

An alternative is to use a weighted sum of the individual proliferation scores. This could be useful, for example, for finding personalized treatments when the distribution of cell types in a tumor is known. When starting weights are used, that objective function tries to minimizes the average proliferation of all cell lines in set C. In the Results section, we will briefly discuss results under this objective. Of course, one could use hybrids of these two objectives as well.

#### Optimizing sequential treatment plans

In the clinic, most anti-cancer drugs are administered in cycles, where drugs are administered for a short period of time, followed by a longer recovery period without treatment. In practice, the same drug is administered in every cycle, but theoretically, it would be possible to use different combinations in each cycle. This is particularly interesting since sub-clones may have different levels of intrinsic resistance or sensitivity to treatment, resulting in distinct drug-dependent growth rates. Therefore, it may not always be possible to find a single treatment that works optimally for all possible sub-clones, and a sequential plan could account for treatment-induced changes to clonal structure. To address this, we also investigate the discovery of a *sequential treatment plan*, that is, a sequence of combination treatments (***τ***_1_, …, ***τ***_*n*_) that is effective on a set of cell lines C. Let the space of sequential treatments $T^{n}\subset R^{7n}$ be the n-ary Cartesian power of the space of drug combinations T. A treatment plan optimization problem is now given by

$$\begin{array}{c} \underset{\left(\tau_{1},\ldots ,\tau_{n}\right)\in T^{n}}{\text{min}}\;\underset{c\in C}{\text{max}}\prod_{i=1}^{n}V_{c}\left(\tau_{i}\right)+\lambda \sum_{i=1}^{n}R\left(\tau_{i}\right). \end{array}$$ (5)

For each cell line c∈C, the relative proliferation value is computed by taking the product of the predicted relative proliferation values at the individual treatment steps. This assumes that the growth of a cell line during one of the steps of the treatment plan multiplicatively affects the growth of that cell line in the next treatment step. A simple, biologically plausible model that satisfies this assumption is an exponential growth model with different, drug-dependent growth rates in each treatment step:

$$\begin{array}{c} V_{c}\left(\eta \left(\tau_{i}\right)\right)=\frac{N_{i-1,\tau ,c}\;\text{exp}\;\left(\eta_{c}\left(\tau_{i}\right)T\right)}{N_{i-1,0,c}\;\text{exp}\;\left(\eta_{c}\left(0\right)T\right)}, \end{array}$$ (6)

where *N*_*i*−1,*τ*,*c*_ is the final cell count of cell line *c* from the previous step in the treated condition, *N*_*i*−1,0,*c*_ is the final cell count of cell line *c* from the previous step *i* − 1 in the untreated condition, *η*_*c*_(*τ*_*i*_) is the treatment-dependent growth rate of cell line *c* during the current step *i*, exp(*η*_*c*_(0)) is the untreated growth rate of cell line *c*, and *T* is the treatment duration (which we assume to be 72 hours, the time used to generate the experimental data the pathway model was calibrated on in prior work). Under the assumption of such an exponential growth model, the following equations hold in every treatment step:

$$\begin{array}{c} V_{c}\left(\tau_{i}\right)=\frac{\text{exp}(\eta \left(\tau_{i}\right)T)}{\text{exp}(\eta (0)T)} \end{array}$$ (7)

and, by induction,

$$\begin{array}{c} \frac{N_{i-1,\tau ,c}}{N_{i-1,0,c}}=\prod_{j=1}^{i-1}V_{c}\left(\tau_{j}\right), \end{array}$$ (8)

assuming that $\frac{N_{0,\tau ,c}}{N_{0,0,c}}=1$, that is, both treated and untreated cell populations start at the same cell counts. This was true for the experimental data used for training the model in prior work.

**Fig 1:**
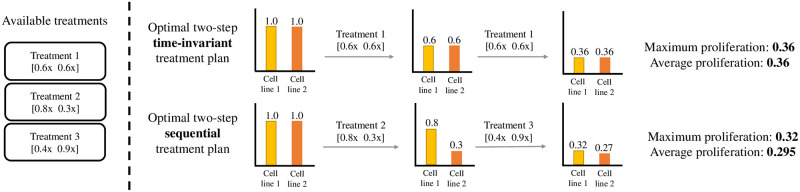
Comparison of a *sequential treatment plan* with a *time-invariant treatment plan*. The use of different specialized drug-combinations (targeting fewer cell lines at once) at different points in time can enable more effective therapies. In the illustrative example above with two cell lines and three available treatments, the optimal two-step time-invariant treatment leads to a relative proliferation score of 0.36 for both cell lines. Meanwhile, the optimal two-step sequential treatment plan achieves relative proliferation scores of 0.32 and 0.27 for cell lines 1 and 2, respectively.

Similar to the multi-cell line setting, this objective function considers the highest proliferation value to find a therapy that is effective for all c∈C. The advantage of sequential plans compared to *time-invariant plans*—plans that use the same drug cocktail in each step of the treatment—is that the use of multiple specialized drug-combinations targeting different subsets of C one at a time can be more effective than a single general τ∈T targeting all of cell lines at once. A small illustrative example for this is shown in Fig 1. In this paper discrete 72h time steps are naturally enforced in that the path-way model is simulated from one steady state to the next.

### Optimization process

The deployed pathway model behaves in a non-convex way when interpolating between drug combinations. Because of this the proposed optimization problems are non-convex and there is no known algorithm that is both scalable and guaranteed to find an optimal solution in every case.

In this work we implemented *covariance matrix adaption evolution strategy* (CMA-ES)—a robust and sample-efficient algorithm [50]. The underlying idea of CMA-ES is to iteratively generate a set of solution candidates whose objective scores are then evaluated. After this, a number of *elites*—that is, the solution candidates with the best objective scores—are selected which are then used to generate the solution candidates for the next iteration step. The CMA-ES algorithm does this by maintaining a mean vector and covariance matrix describing a multivariate Gaussian distribution. At each step, solution candidates are sampled and elites are selected to update the mean and covariance matrix in a way that increases the likelihood of reaching previous elite solution candidates.

Over the years, a large variety of CMA-ES variations have been proposed and applied to various domains. Our implementation of the algorithm exactly follows that presented in [57]. However, we had to make certain modifications to that algorithm to account for the fact that the domain of treatments T is a constrained set with small volume. We will discuss those modifications next.

#### Sampling from a constrained space

During the sampling step, CMA-ES generates a set of solution candidates by sampling from a multivariate Gaussian distribution. When dealing with a constrained domain, naive sampling can lead to the generation of infeasible solution candidates. A popular way to deal with this problem is to simply reject the infeasible points and to sample again until all candidates are feasible [58, 59]. This process effectively transforms the multivariate proposal distributed into a truncated Gaussian.

However, this approach fails in our treatment domain. The volume of domain T roughly shrinks with a factor 1/*d*!, where *d* is the problem dimension. With increasing dimensionality, the vast majority of sampled solution candidates needs to be rejected, rendering the naive rejection-based approach infeasible. While the rejection-based approach took less than one second per iteration to generate candidates for single-step treatment plans, the per iteration time for two-step plans was already up to 5 minutes and for three-step plans we could not complete a single CMA-ES iteration in 8 hours. To avoid this problem, we employ a Hamiltonian Monte Carlo method [60], which can directly generate samples from a truncated multivariate Gaussian distribution that can be constrained by linear and quadratic inequalities. The per iteration sample generation time of this more advanced approach for one-, two- and three-step treatment plans are less than one, three and eight seconds respectively. Thus the Hamiltonian Monte Carlo method can speed up the sample generation process by multiple orders of magnitude. Without this modification extensive simulation studies of *n*-step sequential treatment plans (*d* = 7*n*) would have not been possible.

### Cell lines, penalties, and reference drug combination used in the simulation study

Our simulation study involves 12 colorectal, 19 melanoma, 10 pancreatic, and 20 breast cancer cell lines on which the pathway model was trained in prior work. Cancers from these tissues have a high frequency of BRAF and RAS mutations, for which a large fraction of drugs in the model is thought to be effective. As mentioned in the objective definition, a penalty parameter λ is used to trade-off between reduction in predicted proliferation and adverse effects as quantified by the regularization functions. Because it is not clear how to choose a single best λ ahead of time, we varied the penalty parameter λ from 10^−7^ to 10^−1^ with exponent steps of 0.25 (0.05 for the sequential treatments) which recovered a set of Pareto efficient treatment candidates. Empirically, we observed that 10^−1^ provides a natural upper bound so conservative that the algorithm decides to not administer any treatment at all. A lower bound of 10^−7^ allowed the algorithm to select the most aggressive dosage of 8000 nM and the treatment compositions for all regularizers were very similar. While in most cases the optimization process converged in less than 100 iterations, we ran the algorithm for 400 iterations because we observed large variance in the returned treatments when using logarithmic regularization with large λ values indicating a difficult optimization surface. Regarding the hyperparameters we exactly follow the CMA-ES implementation discussed in the book by Kochenderfer and Wheeler [57]. For single/two-step plans we sample 9/11 candidates per iteration and select 4/5 elites. Candidate generation variance is *σ* = 0.25. The initial starting point is the treatment that splits 8000 nM evenly among the 8 drugs. For every problem configuration, the optimization algorithm is initialized with 3 different random seeds and the search result with best objective function value is reported.

We compare the optimized treatments to two baselines. The first baseline is the best single-drug treatment which is determined as follows. For each of the 7 drugs, treatments using concentrations in the range from 0 nM to 8000 nM are considered (8000 nM matches the maximal concentration in the clinical data the ODE model was fitted with). Their objective and relative proliferation values are evaluated at 1 nM steps. For a given penalty parameter λ, the best single drug treatment is identified by its objective value. The second baseline are two-drug combinations that use a mixture of PLX-4720 (RAFi)+PD0325901 (MEKi). PLX-4720 and PD0325901 serve as a proxy for the clinical grade combination therapy of Vemurafenib (RAFi) and Cobimetinib (MEKi) for BRAF mutant melanoma [5]. Vemurafenib is the clinical analogue of PLX-4720 and PD0325901 and Cobimetinib are allosteric inhibitors that target similar pockets in MEK molecules. As it was difficult to find precise information on the clinical mixture ratios for these two drugs, we consider ratios from 0%-100% evaluated at 5% steps. As for the single drug baseline, treatments that use a total concentration in the range from 0 nM to 8000 nM are evaluated at 1 nM steps, and the two-drug treatment that achieves the best objective value is used as the second baseline.

### Computation

All simulations were conducted using a compute cluster. Each individual optimization was run on a single 64-core server with AMD Opteron(TM) 6272 2.1 GHz processors and required less then 64 GB of RAM. Each prediction of proliferation for a given cell line and treatment (that is, one call to the function *V*_*c*_) took about 1 second. This dominated the run-time of the CMA-ES algorithm. We parallelized the evaluation of treatment candidates generated by the CMA-ES algorithm, and furthermore, for each solution candidate, parallelized the evaluation of that treatment on the different cell lines. In this way, we were able to run all simulations in less than two weeks.

## Results

In this section we evaluate the effectiveness of the combination therapy candidates identified by the modified CMA-ES algorithm for the three treatment settings. For each setting, the findings are illustrated and the resulting treatments are compared to the two baselines. We also analyze the variance of the returned treatments as well as optimization for average proliferation.

### Optimizing the single-step treatment of a single cell line

**Fig 2:**
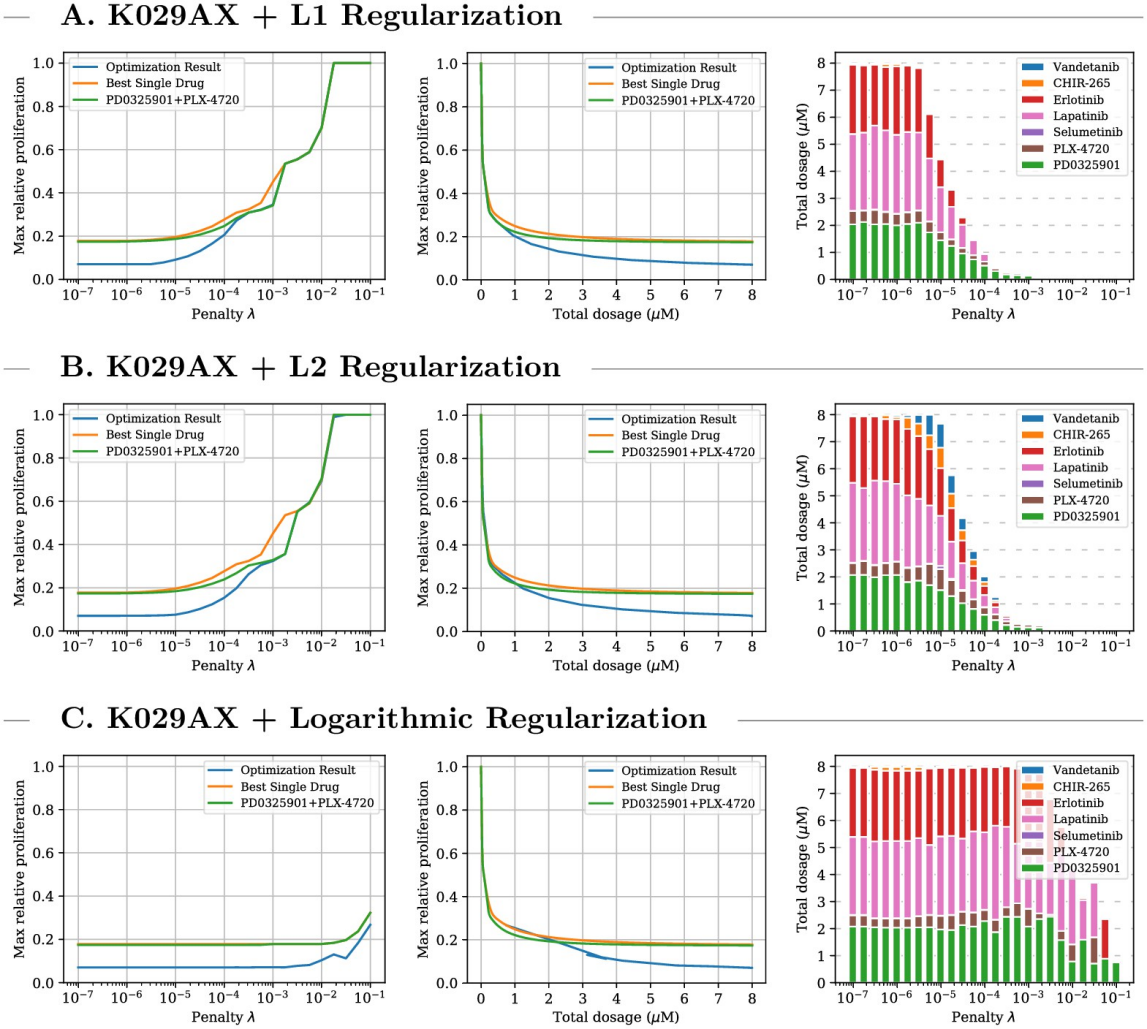
Single-step treatment for K029AX. Comparison between optimized single-cell multi-drug treatment, optimal single-drug treatment, and optimal PD0325901/PLX-4720 combination treatment for K029AX—a melanoma cell line with BRAF V600E mutation—for three different types of regularization. Left plots: optimal treatment as identified by the objective function for different penalty parameters. The middle plots: relationship between administered total dosage and achieved proliferation value regardless of penalty and objective value. Right plots: composition of the multi-drug treatments. For all three types of regularization the optimization process leads to combination treatments which achieve significantly lower predicted proliferation values at lower concentrations than single and two-drug treatment. The treatment composition varies with the type of regularization.

In the first setting, the objective function defined by Eq (3) is used to find effective drug-combinations for individual cell lines. Fig 2 visualizes the optimization results for K029AX—a melanoma cancer with BRAF V600E mutation—for three different types of regularization. The optimized treatments achieve substantially lower relative proliferation values at lower total dosage than the two baseline treatments showing the suitability of the CMA-ES algorithm. For low penalties all regularizations lead to similar treatment compositions. For higher penalties L2 regularization leads to treatments that use more drugs at lower dosage and logarithmic regularization leads to treatments that use fewer drugs at higher dosage. L1 regularization penalizes only the total dosage and is agnostic towards the specific treatment composition. Thus a treatment that is optimal under L1 regularization achieves the strongest possible reduction in cancer cell proliferation with respect to its total dosage. Given two-drug combinations that employ the same total dosage and that achieve the same treatment effect, L2 regularization prefers the one that balances its components more evenly. Logarithmic regularization penalizes treatments that employ multiple drugs harshly and a low objective value does not always identify a strong treatment (this might be explained with variance in the optimization process which we analyze later on). The middle column of Fig 2 visualizes Pareto efficient treatments and can be used to trade-off proliferation rate reduction with total administered dosage. Further results for A2058 and MDAMB435S—two other melanoma cancer with BRAF V600E mutation—are provided in S1 Appendix. For A2058 the optimized combination treatments are substantially more efficient than the baselines for all dosages over 500 nM. For MDAMB435S, the clinical-grade combination therapy that uses PLX-4720 and PD0325901 is already very effective and the discovered treatment only leads to slight improvements. This can be seen as evidence that the clinical-grade therapy is close to optimal in the space of possible multi-drug treatments captured by the ODE model.

### Optimizing the single-step treatment of a population of cell lines

**Fig 3:**
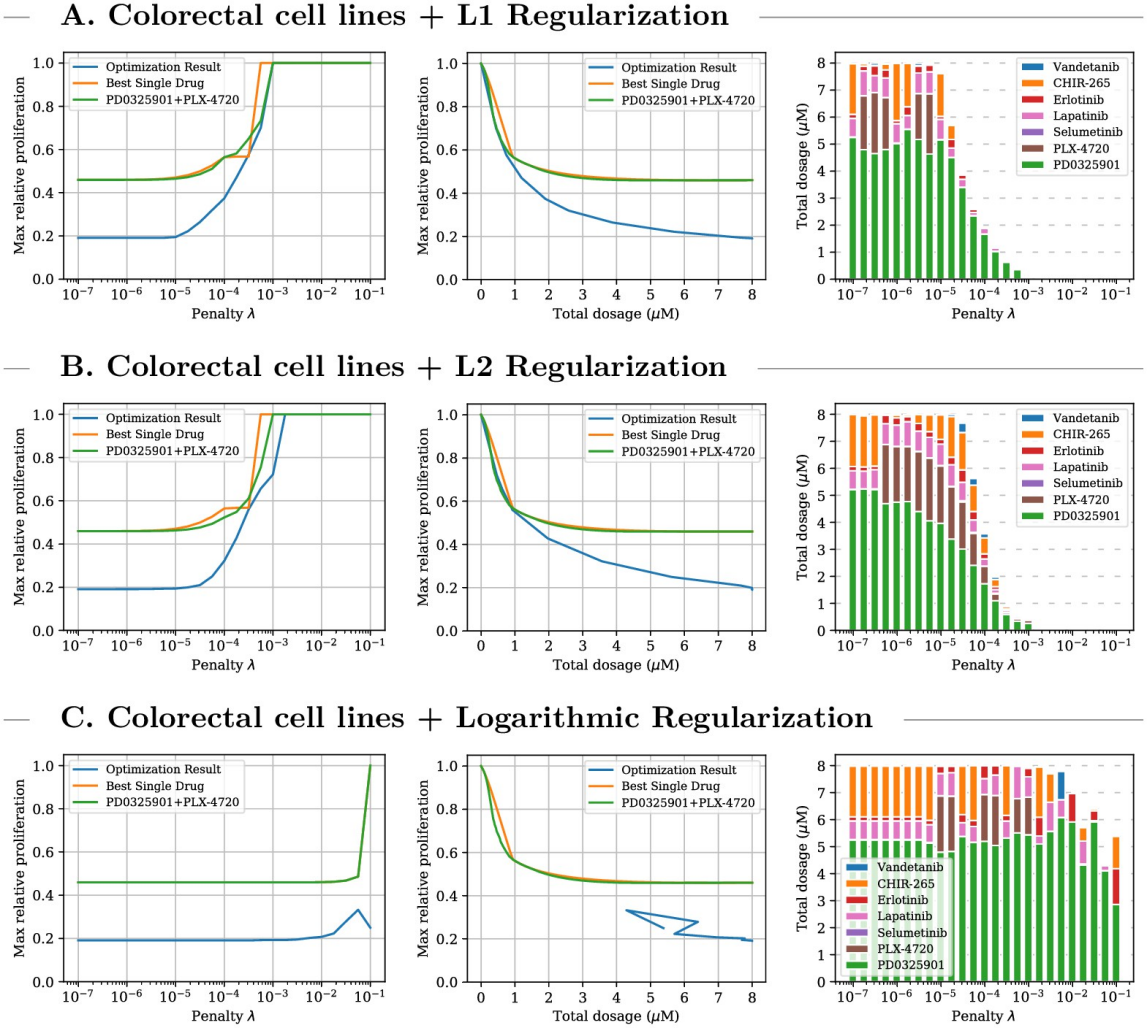
Single-step treatment for colorectal cell lines. Comparison between optimized multi-cell multi-drug treatment, optimal single-drug treatment, and optimal PD0325901/PLX-4720 combination treatment for colorectal cell lines for three different types of regularization. Left plot: optimal treatment as identified by the objective function for different penalty parameters. Middle plot: relationship between administered total dosage and achieved proliferation value regardless of objective values. Right plot: composition of the multi-drug treatments. For all three types of regularization the optimization process leads to combination treatments which achieve significantly lower predicted proliferation values at lower concentrations than single and two-drug treatment. When using the logarithmic regularization low objective values did not always indicate favorable proliferation values.

For the second setting, the objective function defined by Eq (4) is used to find drug combinations that minimize the maximum relative proliferation value predicted by the pathway model over sets of cell lines originating from skin ($C_{\text{Melanoma}}$), large-intestine ($C_{\text{Colorectal}}$), pancreas ($C_{\text{Pancreatic}}$), and breast ($C_{\text{Breast}}$) tissues, respectively. Findings for colorectal cell lines are visualized in Fig 3. Results for the three other tissues under all three types of regularization are provided in S2 Appendix. For all four tissues, the discovered treatments achieve substantially lower maximum relative proliferation values than the single-drug and PD0325901/PLX-4720 combination baselines at medium and high dosages. Especially for pancreatic cell lines, the optimized treatments reduce the predicted cancer cell viability by a factor of up to three for penalty values λ < 10^−4.5^ which allow the algorithm to employ the maximum (most aggressive) dosage of 8000 nM. For breast cancers, the optimization process leads to drug combinations that achieve notable treatment effects even at dosages below 500 nM. The predicted advantage of combination therapies over the baselines in the multi-cell regime is even more pronounced than in the single-cell regime.

### Optimizing sequential treatment plans

**Fig 4:**
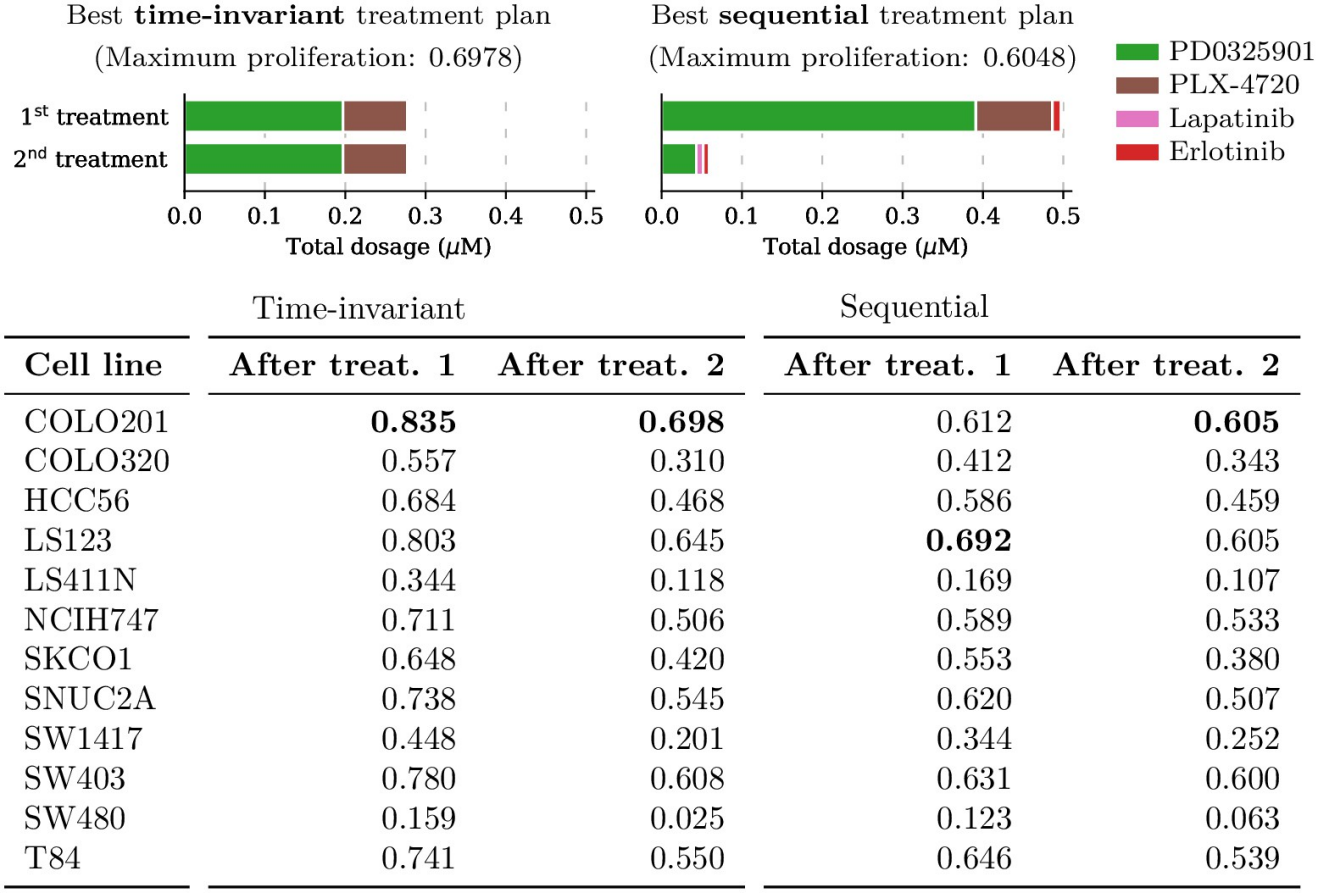
Two-step treatment plan composition for colorectal cell lines. A visualization of the drug cocktails administered by the optimized two-step treatment plan and the optimized two-step time-invariant treatment plan for colorectal cell lines under L1 regularization at the same total drug dosage (550 nM). The 2-step plan uses a high-dose treatment followed by a low-dose treatment. This achieves maximum proliferation 0.6048, which is more effective than the time-invariant treatment plan which only achieves 0.6978.

The third setting investigates 2-step treatment plans and uses the objective function defined by Eq (5) to find sequences of drug combinations that are effective on cell lines originating from the same tissue. In this setting we compare the performance of optimized sequential treatment plans (that is, ones that can use different drug combinations and dosages at the two treatment steps) described by 14 variables against optimized time-invariant treatment plans (that is, ones that have to use the same drug combination and dosage in each of the two treatment steps) described by 7 variables. With these candidate drugs and cell lines, only very slight benefits were gained from allowing time-varying treatments. However, in a few cases at medium dosages we observed some larger gains. One example for an effective 2-step plan for colorectal cell lines is shown in Fig 4. A sequential plan that first employs one more aggressive drug-combination of PD0325901, PLX-4720, and Erlotinib and then a more conservative—that is, lower-dose—combination of PD0325901, Lapatinib, and Erlotinib achieves a maximum predicted proliferation value of 0.6048 which is 13% lower than the proliferation value achieved by the optimized time-invariant treatment plan (0.6978), which uses the clinical-grade drug pair of PD0325901 and PLX-4720 at medium dosage.

### Optimization for average proliferation

While this paper mainly focuses on minimizing the maximum proliferation value in populations of cancer cell lines, we will now briefly discuss optimization results where the objective was to minimize the average proliferation value. This alternative optimization problem was discussed in the methods section.

First we investigated what average proliferation rate the multi-cell combination treatments that were optimized for the *maximum* criterion achieve for each of the four individual tissues under L1 regularization with penalty value λ = 10^−7^. We found that the treatments optimized for low maximum proliferation achieve an average relative proliferation rate of approximately 0.1036 across melanoma, 0.0814 across colorectal, 0.1019 across pancreatic, and 0.2010 across breast cancer cell lines. We compared these scores to those attained by the multi-cell combination treatments which were specifically designed to minimize the *average* proliferation rate. We found that the treatments optimized for low average proliferation rate achieve average predicted proliferation values of approximately 0.0816 across melanoma, 0.0712 across colorectal, 0.0804 across pancreatic, and 0.1589 across breast cancers. Therefore, the multi-cell treatments considered in this paper not only minimize the maximum proliferation rate of cells originating from each tissue type, but they also attain average proliferation rates that are within 20% of what is attained by the treatments which were specifically designed for low average. A potential explanation for this behaviour is that both objectives lead to treatments focusing on the same cell lines. The cell-specific proliferation rates shown in Fig 4 reveal that there are a few cell lines which a much harder to treat than others. Any drug combination that wants to achieve low average or maximum proliferation needs to focus on these few hard-to-treat cell lines.

### Variance in optimization process

During our single- and multi-cell simulations we observed some variance in the optimized combination treatments when using low penalty values. For example in Fig 3, there appears to be a non-smooth dependency of PLX-4720 and CHIR-265 concentrations on the value of λ. As PLX-4720 and CHIR-265 both have RAF as primary target with similar affinity, the two drugs can be applied interchangeably without larger effect on the objective function and penalty term. This might induce an indeterminacy in the optimization problem and that the optimization runs convergence to multiple distinct local optima, causing the non-smooth dependence on λ. To get a better insight into this behavior we performed additional single-cell optimization runs with K029AX as well as multi-cell optimizations with colorectal cell lines. For both settings we ran an additional 20 runs with warm starts. Each run started by optimizing a treatment for the lowest penalty value (λ = 10^−7^) and then increased the penalty exponent at 0.25 steps, where at each step we initialized the algorithm with the optimal drug-combination from the previous step.

**Fig 5:**
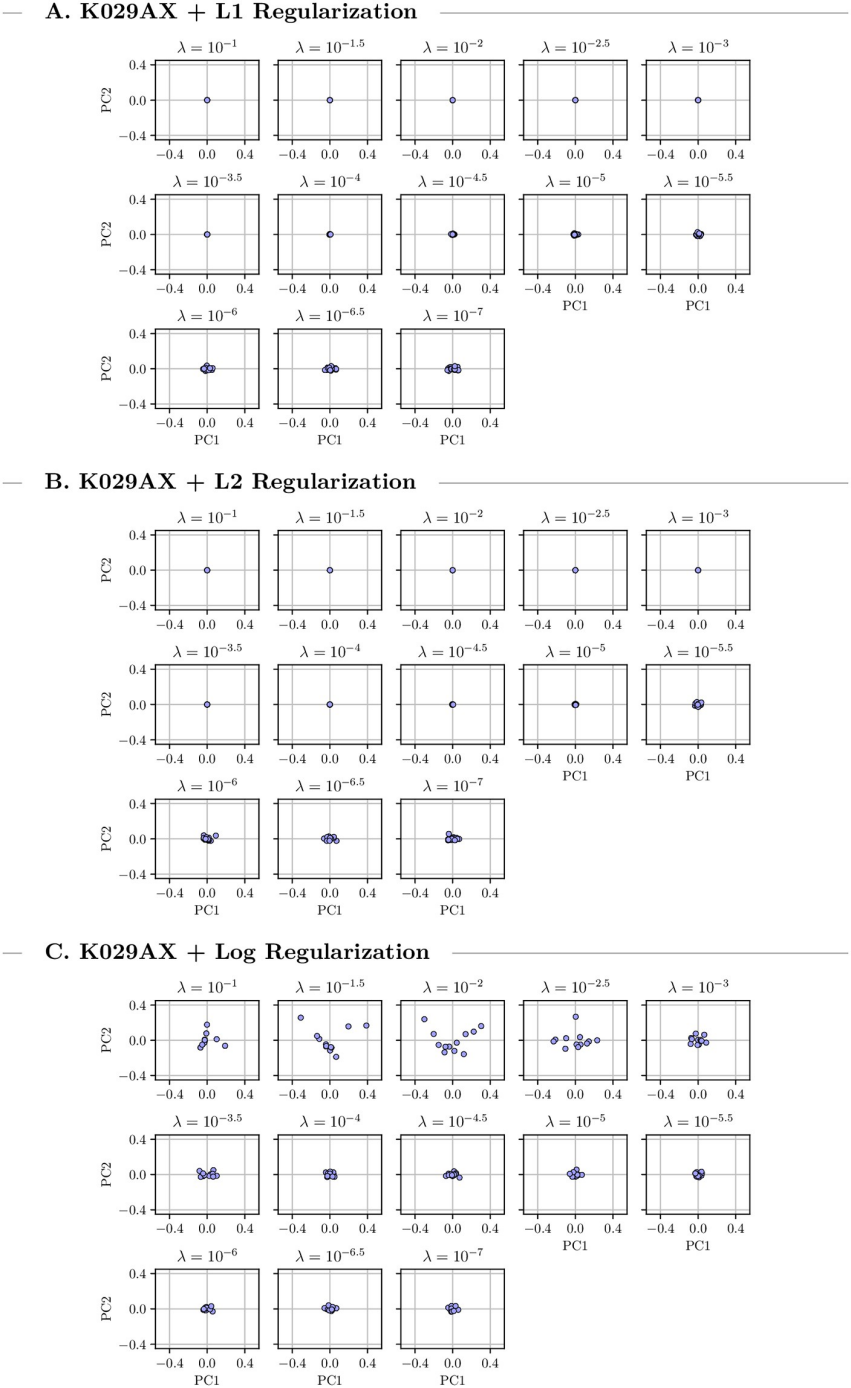
Output variance for K029AX. Visualization of the first two principal components of 20 single-cell combination treatments for K029AX under three different types of regularization using warm starts. The treatment variance varies with changing penalty parameter.

**Fig 6:**
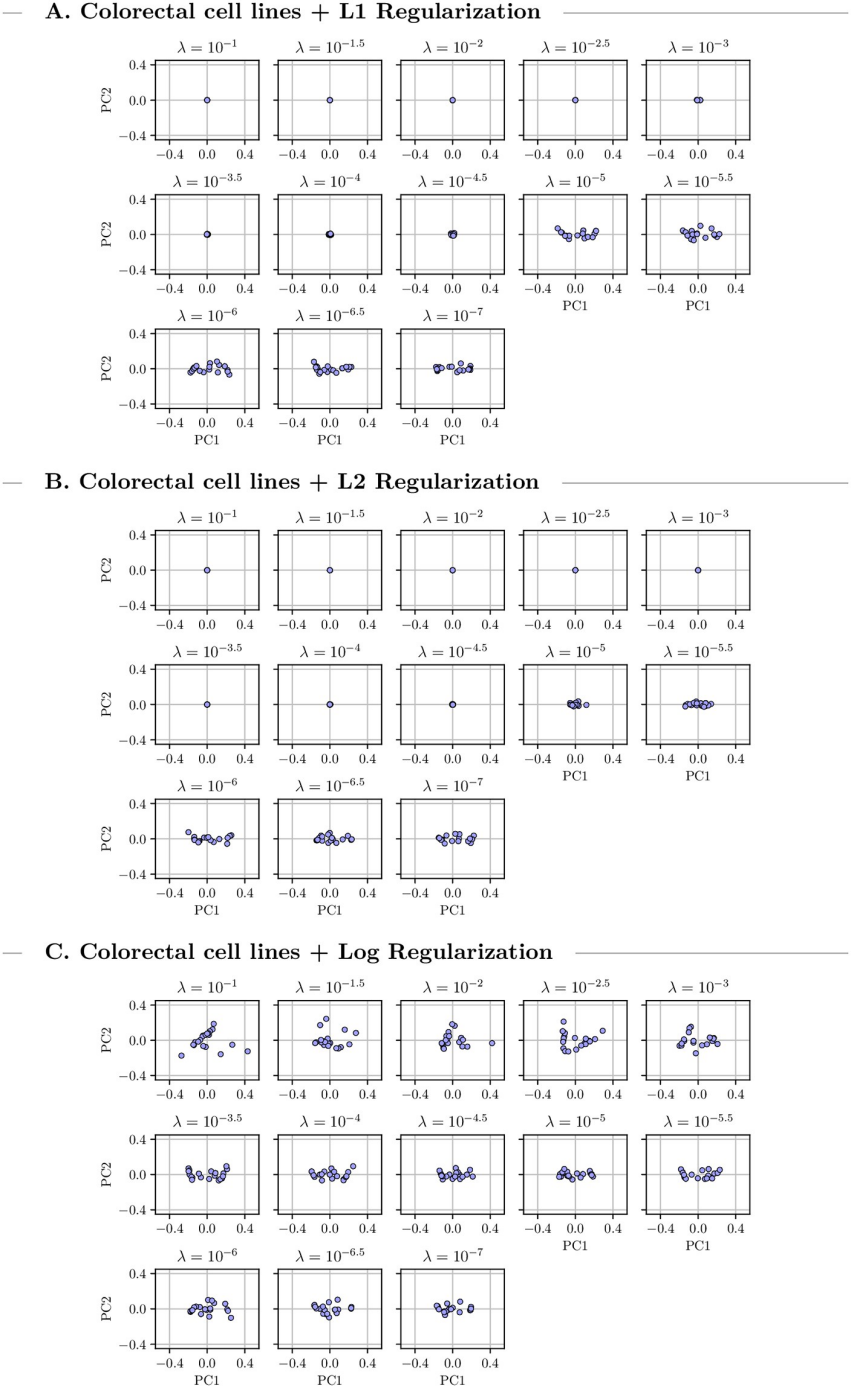
Output variance for colorectal cells. Visualization of the first two principal components of 20 multi-cell combination treatments for colorectal cells under three different types of regularization using warm starts. The treatment variance varies with changing penalty parameter.

We grouped the discovered drug-combinations found during the 20 runs by penalty value and performed separate Principal Component Analysis (PCA) for each group to investigate the treatment distribution. The first two principal components are visualized in Figs 5 and 6 which in both setups explained more than 90% of the existing variance. Under high to medium penalties L1 and L2 regularization led to unique optimal treatments. For lower penalty values there is some variance. Logarithmic penalization suffers from high variance even when using large penalties indicating multiple local optima. This might explain some of the instabilities we observed in the previous CMA-ES runs which used logarithmic regularization. For low penalty values the distribution of the returned combinations is similar for all types of regularization which indicates stable convergence of the optimization algorithm. Overall the variance in the multi-cell optimizations is larger than in the single-cell one.

## Discussion

Our approach discovered treatment candidates that deviate from current clinical first-line treatment strategies and are predicted to achieve larger reductions in cancer cell proliferation. Yet, we have to carefully examine whether the proposed strategies are plausible from a biological perspective. For the investigations with BRAFV600E skin cancer cell lines, the optimal combination strategy we identified was often only marginally better than the PD03525901+PLX-4720 gold-standard reference. Similarly, for the multi-cell line analysis, the algorithm identified the gold-standard combination for low total dosages and was only able to identify better combinations at higher dosages. However, we consistently observed high concentrations of the MEK inhibitor PD0325901, which is known to display otherwise rare on-target toxicities, suggesting that a different regularization strategy might be desired for this drug.

Another question is how likely it is that the treatments which are predicted to be effective by the pathway model will also be effective in a real wet lab study. Overfitting is a concern when calibrating a model with over 4000 parameters. We used a trained mechanistic pathway model that was fitted in prior work [9] with over 5000 real data points from the Cancer Cell Line Encyclopedia [19]. Unlike in many other systems biology settings here the number of data points is larger than the number of model parameters. The model itself and its calibration process are described in great detail in [9]. One advantage of mechanistic models is that their graphical structure captures domain knowledge of the underlying cell biology. This makes mechanistic models less prone to overfitting and can help with generalization. The used pathway model was calibrated with data from single-drug treatments and has been shown to accurately predict treatment effects of two-drug combination treatments [9]. Given that most of the optimized treatments only use 3 or 4 drugs, we are optimistic that the model can yield valuable insights. Nonetheless, laboratory studies are, of course, required to provide a final answer about the effectiveness of the treatment candidates identified here.

One limitation of the current study is that the relative cell viability measures we have used here, such as those reported by assays such as CellTiter-Glo, are subject to several known inconsistencies [61, 62]. These issues can, in part, be addressed by more modern methods [63, 64]. Similarly, the assumption that cell growth dynamics have reached a steady state after 72 hours may not always hold true. This may influence whether and how well biological insights presented in this study can be replicated in *in vitro* and *in vivo* experiments. However, these limitations are primarily due to limitations of data available in the large pharmacological studies [19] that were used in the parameterization of the current model, and not due to intrinsic shortcomings of the methods developed in this study. In fact, the methods developed here could easily be applied to the design of adaptive treatment strategies [16].

The model employed here assumes cell-line-specific, but static transcription. Accordingly, the model may not accurately describe adaptive resistance mechanisms that are believed to work through transcriptional feedbacks [20, 65]. Moreover, because the steady state of the model is always unimodal under conditions we have considered, there is no memory effect between subsequent treatments at the cellular level. However, the multiplicative propagation of relative viabilities along the sequence of treatments introduces a memory effect at the population level. In every treatment step, the relative proliferation values from the previous step effectively introduce a re-weighting of the relative importance of the cell lines. As we showed, this alone is enough to cause there to be benefit from time-varying sequential treatments. In practice, a further benefit from sequential treatment may be obtainable by steering a cell line or set of cell lines during the dynamics, that is, without waiting for steady state between treatments. Finding such treatment plans computationally would require a signaling pathway model that is faithful to reality not only at steady states but also during the transient paths. Constructing and calibrating such models would likely require significantly more *in vivo* and/or *in vitro* data than models that only need to be accurate in steady states. The approach we present here itself does not make assumptions about the properties of the underlying model or what biological processes it describes. In particular, as the employed optimization approach is a gradient-free method, it could also be applied to, for example, stochastic agent-based models.

For some cell-lines and regularizers, we observed that optimization can yield a continuum of equivalent optimal treatments, which indicates ill-conditioning of the problem. Looking at the PCA (Figs 5 and 6) revealed that this behavior is limited to low penalization strengths that do not reduce the total concentration of the optimal treatment beyond the 8 *μM* maximum. Accordingly, we concluded that this ill-conditioning did not substantially effect the results presented here and that the regularization approaches, as expected, improved the conditioning of the problem.

The regularization functions we used provide an empirical way to minimize drug concentrations and respective adverse toxicities. In practice, concentrations at which adverse toxicities occur may be specific to drugs, tissues, and individuals. In the absence of large-scale toxicological and pharmacokinetic screenings, it seems difficult to design a more rational type and strength of penalization. Our regularization functions penalize total drug burden and do not consider cooperativity. The study of drug cooperativity is in itself an active area of research [66–71].

## Conclusions

In this paper we proposed a framework for *in silico* combination treatment optimization. To the best of our knowledge this is the first time a large-scale pan-cancer pathway model was used to identify candidates for effective combination therapies. Multiple treatment optimization problems were proposed which required us to balance reduction in proliferation with adverse side effects. In order to solve these problems, we combined the CMA-ES algorithm with a significantly more scalable sampling scheme, based on a Hamiltonian Monte-Carlo method. We evaluated the approach in an extensive simulation study of cancer cell lines originating from multiple tissues. We studied the treatment of individual cell lines and heterogeneous populations of cell lines. We also studied the generation of sequential time-varying and time-invariant treatment plans. The combination treatment candidates identified by our algorithm achieved significantly better predicted proliferation scores at lower drug concentrations compared to the conventional therapy approaches. This serves as an early proof of concept of how *in silico* simulations can be used to identify potentially novel combination therapies. Future research is required to evaluate the performance of the discovered treatments in laboratory studies.

## Supporting information

S1 Appendix Further single-step single-cell simulations. Results of the single-step single-cell optimization process for A2058 and MDAMB43S cancer cell lines. For A2058 we observed that for all three types of regularization the optimized combination treatments achieve significantly lower relative proliferation values at lower concentrations than the single-drug and two-drug baselines. For MDAMB43S the discovered combination treatments only slightly improved upon the PD0325901/PLX-4720 two-drug baseline. In both cases the type of regularization impacts the composition of the returned combination treatments. When using logarithmic regularization we observed large variance in returned treatments and low objective values did not always indicate effective treatments.
(PDF)

S2 Appendix Further single-step multi-cell simulations. Results of the single-step multi-cell optimization process for melanoma, pancreatic and breast cancer cell lines. For all three tissues and regularizers, the discovered combination treatments achieve significantly lower maximum relative proliferation values than the single-drug and PD0325901/PLX-4720 combination baselines at medium and high dosages. Especially for pancreatic cell lines, the optimized treatments reduce the cancer cell viability by a factor of more than two. For breast cancers, the optimization process leads to drug combinations that achieve notable treatment effects even at low dosage. The type of used regularization effects the composition of the combinations. When using logarithmic regularization we observed large variance in returned treatments and low objective values did not always indicate effective treatments.
(PDF)
