# Supplementary material for: Combination treatment optimization using a pan-cancer pathway model
Source: PLoS Comput Biol. 2021 Dec 28;17(12):e1009689. doi: 10.1371/journal.pcbi.1009689 (PMC8747684; doi:10.1371/journal.pcbi.1009689)
Supplement: S1 Appendix — Results of the single-step single-cell optimization process for A2058 and MDAMB43S cancer cell lines. For A2058 we observed that for all three types of regularization the optimized combination treatments achieve significantly lower relative proliferation values at lower concentrations than the single-drug and two-drug baselines. For MDAMB43S the discovered combination treatments only slightly improved upon the PD0325901/PLX-4720 two-drug baseline. In both cases the type of regularization impacts the composition of the returned combination treatments. When using logarithmic regularization we observed large variance in returned treatments and low objective values did not always indicate effective treatments. (PDF) [file pcbi.1009689.s001.pdf]

S1 Further Single-Step Single Cell Simulations

We show results of the single-step single-cell optimization process for A2058 (Fig A) and MDAMB43S (Fig B) cancer cells. For A2058 we observed that for all three types of regularization the optimized combination treatments achieve significantly lower predicted proliferation values at lower concentrations than the single and two-drug baselines. For MDAMB43S the discovered combination treatments only slightly improved upon the PD0325901/PLX-4720 two-drug baseline. In both cases the type of regularization impacts the composition of the returned combination treatments. When using logarithmic regularization we observed large variance in the treatments and low objective values did not always indicate effective treatments.

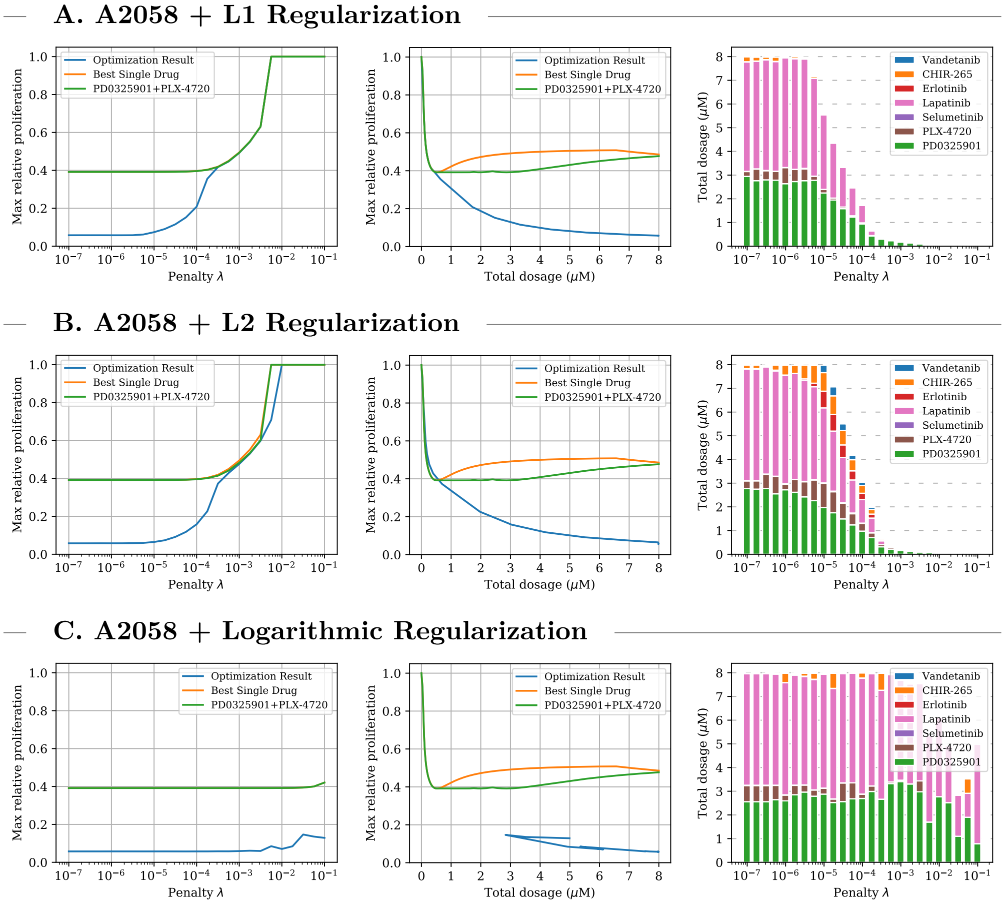

Fig A. Single-step treatment for A2058. Comparison between optimized single-cell multi-drug treatment, optimal single-drug treatment, and optimal PD0325901/PLX-4720 combination treatment for A2058—a melanoma cell line with BRAF V600E mutation—for three different types of regularization. Left plots: optimal treatment as identified by the objective function for different penalty parameters. The middle plots: relationship between administered total dosage and achieved proliferation value regardless of penalty and objective value. Right plots: composition of the multi-drug treatments.

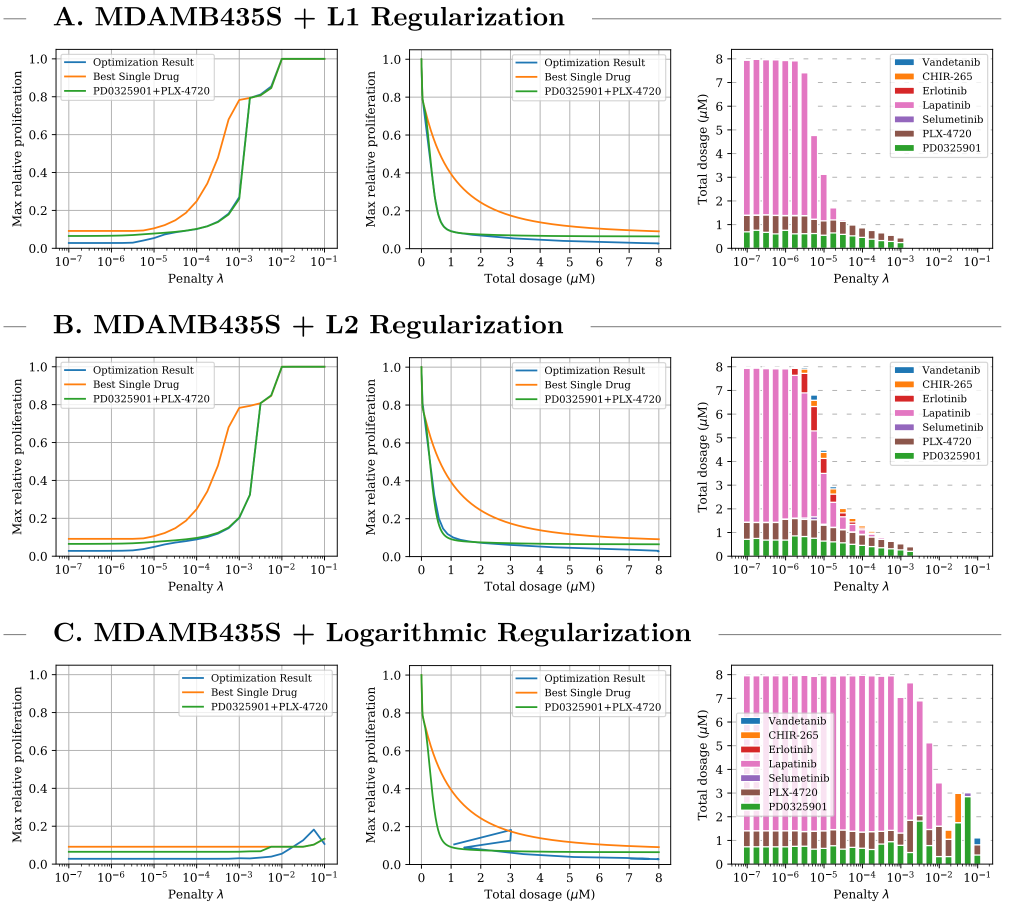

Fig B. Single-step treatment for MDAMB435S. Comparison between optimized single-cell multi-drug treatment, optimal single-drug treatment, and optimal PD0325901/PLX-4720 combination treatment for MDAMB435S—a melanoma cell line with BRAF V600E mutation—for three different types of regularization. Left plots: optimal treatment as identified by the objective function for different penalty parameters. The middle plots: relationship between administered total dosage and achieved proliferation value regardless of penalty and objective value. Right plots: composition of the multi-drug treatments.
