# Supplementary material for: Combination treatment optimization using a pan-cancer pathway model
Source: PLoS Comput Biol. 2021 Dec 28;17(12):e1009689. doi: 10.1371/journal.pcbi.1009689 (PMC8747684; doi:10.1371/journal.pcbi.1009689)
Supplement: S2 Appendix — Results of the single-step multi-cell optimization process for melanoma, pancreatic and breast cancer cell lines. For all three tissues and regularizers, the discovered combination treatments achieve significantly lower maximum relative proliferation values than the single-drug and PD0325901/PLX-4720 combination baselines at medium and high dosages. Especially for pancreatic cell lines, the optimized treatments reduce the cancer cell viability by a factor of more than two. For breast cancers, the optimization process leads to drug combinations that achieve notable treatment effects even at low dosage. The type of used regularization effects the composition of the combinations. When using logarithmic regularization we observed large variance in returned treatments and low objective values did not always indicate effective treatments. (PDF) [file pcbi.1009689.s002.pdf]

S2 Further Single-Step Population Cell Simulations

We show results of the single-step multi-cell optimization process for melanoma (Fig A), pancreatic (Fig B) and breast (Fig C) cancer cell lines. For all three tissues and regularizers, the discovered combination treatments achieve significantly lower maximum predicted proliferation values than the single-drug and PD0325901/PLX-4720 combination baselines at medium and high dosages. Especially for pancreatic cells, the optimized treatments reduce the viability score by a factor of more than two. For breast cancers, the algorithm lead to drug combinations that achieve notable treatment effects even at low dosages. The type of used regularization effects the composition of the combinations. When using logarithmic regularization we observed large variance in the treatments and low objective values did not always indicate effective treatments.

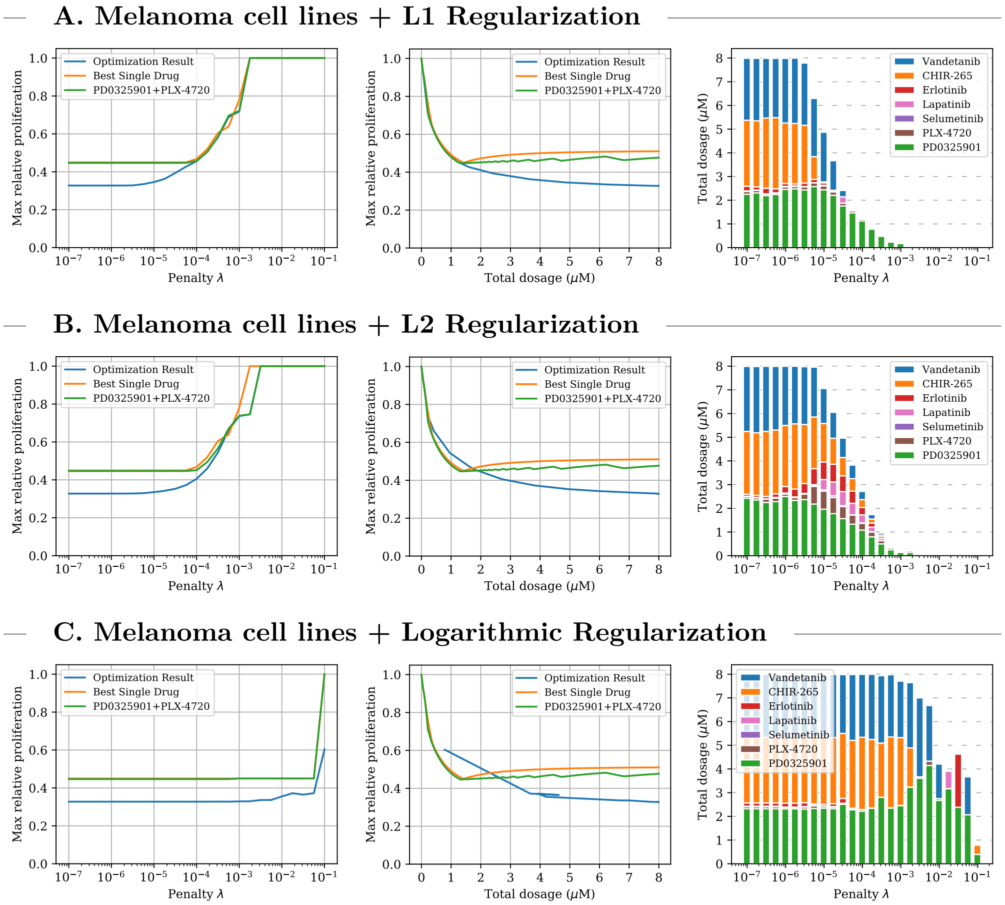

Fig A. Single-step treatment for melanoma cell lines. Comparison between optimized multi-cell multi-drug treatment, optimal single-drug treatment, and optimal PD0325901/PLX-4720 combination treatment for melanoma cell lines for three different types of regularization. Left plot: optimal treatment as identified by the objective function for different penalty parameters. Middle plot: relationship between administered total dosage and achieved proliferation value regardless of objective values. Right plot: composition of the multi-drug treatments.

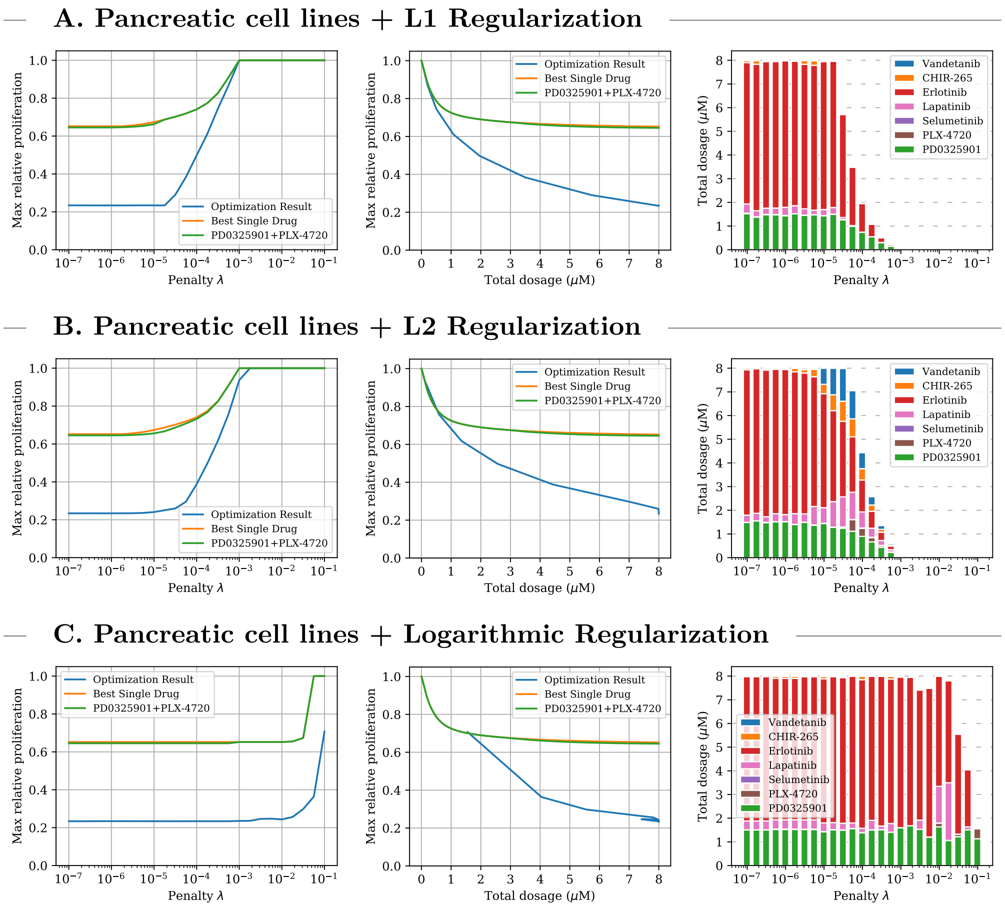

Fig B. Single-step treatment for pancreatic cell lines. Comparison between optimized multi-cell multi-drug treatment, optimal single-drug treatment, and optimal PD0325901/PLX-4720 combination treatment for pancreatic cell lines for three different types of regularization. Left plot: optimal treatment as identified by the objective function for different penalty parameters. Middle plot: relationship between administered total dosage and achieved proliferation value regardless of objective values. Right plot: composition of the multi-drug treatments.

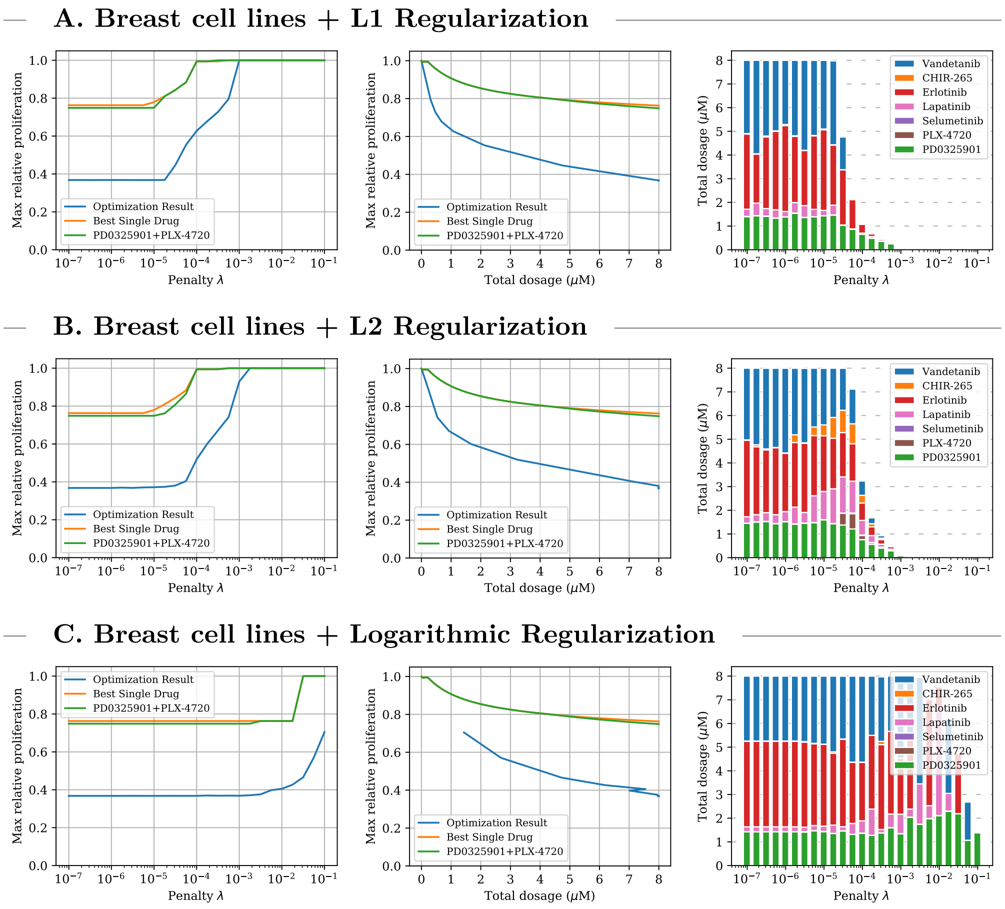

Fig C. Single-step treatment for breast cell lines. Comparison between optimized multi-cell multi-drug treatment, optimal single-drug treatment, and optimal PD0325901/PLX-4720 combination treatment for breast cancer cell lines for three different types of regularization. Left plot: optimal treatment as identified by the objective function for different penalty parameters. Middle plot: relationship between administered total dosage and achieved proliferation value regardless of objective values. Right plot: composition of the multi-drug treatments.
